# A Comparative Mini-Review on Transition Metal Oxides Applied for the Selective Catalytic Ammonia Oxidation (NH_3_-SCO)

**DOI:** 10.3390/ma15144770

**Published:** 2022-07-07

**Authors:** Magdalena Jabłońska, Alejandro Mollá Robles

**Affiliations:** Institute of Chemical Technology, Universität Leipzig, Linnéstr. 3, 04103 Leipzig, Germany; alejandro.molla_robles@uni-leipzig.de

**Keywords:** selective ammonia oxidation, hydrotalcite-like compounds, mixed metal oxides, transition metals

## Abstract

The selective catalytic oxidation of NH_3_ (NH_3_-SCO) into N_2_ and H_2_O is an efficient technology for NH_3_ abatement in diesel vehicles. However, the catalysts dedicated to NH_3_-SCO are still under development. One of the groups of such catalysts constituted transition metal-based catalysts, including hydrotalcite-derived mixed metal oxides. This class of materials is characterized by tailored composition, homogenously dispersed mixed metal oxides, exhibiting high specific surface area and thermal stability. Thus, firstly, we give a short introduction to the structure and composition of hydrotalcite-like materials and their applications in NH_3_-SCO. Secondly, an overview of other transition metal-based catalysts reported in the literature is given, following a comparison of both groups. The challenges in NH_3_-SCO applications are provided, while the reaction mechanisms are discussed for particular systems.

## 1. Introduction

**Figure 1:**
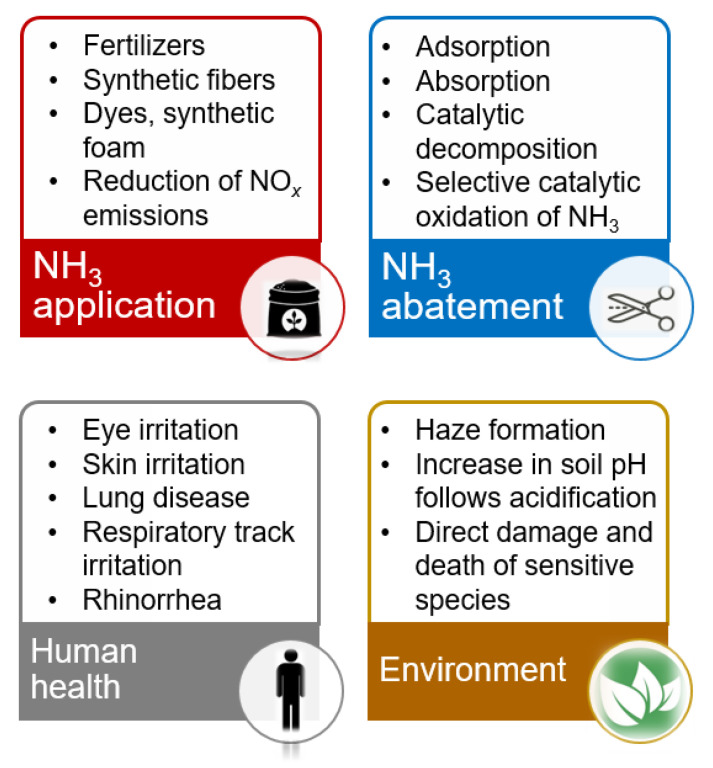
Schematic representation of NH_3_ application and abatement, as well as the impact of NH_3_ on human health and the environment.

Ammonia (NH_3_) is one of the most important chemicals in the world, e.g., used to produce fertilizers, synthetic fibers, dyes and synthetic foam, as well as to reduce NO*_x_* emissions, etc. (Figure 1). However, since 2001, the EU has listed NH_3_ as one of the four main types of atmospheric pollutants (among NO*_x_*, SO_2_, and non-methane volatile organic compounds (NMVOC)), and has released the EU National Pollutant Discharge Inventory (NECD) every year. Twelve Member States (including, e.g., Germany, France, Austria, etc.), and the United Kingdom need to reduce NH_3_ emissions by up to 10% against 2018 levels to attain their 2020 and 2030 emission reduction commitments. Denmark and Lithuania need to reduce emissions by more than 10% [1]. Ammonia emitted from livestock, industrial processes or NH_3_ emitted to the atmosphere through either the large-scale usage of fertilizers or gas slippage from the NH_3_-SCR-DeNO*_x_* applications can cause serious damage to human health (i.e., to the eyes, throat, nose, etc., if its concentration exceeds 50–100 ppm) [2], and environment (e.g., acidification, formation of haze, Figure 1).

To control the ammonia slip, several different techniques used for the elimination of NH_3_ (e.g., adsorption, absorption, catalytic decomposition, etc.) have been applied [3]. However, the selective catalytic oxidation of ammonia into nitrogen and water vapor is an ideal technology for removing NH_3_ from O_2_-containing waste gases, after the selective catalytic reduction of NO*_x_* by NH_3_ (SCR-DeNO*_x_*, by the use of stoichiometric or even excess amount of NH_3_) from stationary and mobile sources. Thus, ideally, the residual NH_3_ (slip) could be selectively oxidized to N_2_ and H_2_O (i.e., inert, and non-toxic products, Equation (1). However, the N_2_ selectivity is affected by the undesired oxidation of ammonia to N_2_O, NO, and NO_2_ (Equations (2)–(4)):

4NH_3_ + 3O_2_ → 2N_2_ + 6H_2_O (1)

2NH_3_ + 2O_2_ → N_2_O + 3H_2_O (2)

4NH_3_ + 5O_2_ → 4NO + 6H_2_O (3)

4NH_3_ + 7O_2_ → 4NO_2_ + 6H_2_O (4)

Thus, NH_3_-SCO catalysts with enhanced activity, N_2_ selectivity, stability (also in the presence of H_2_O, SO*_x_*, and CO*_x_*, up to 600–700 °C in the cycle of diesel particulate filter regeneration) and low cost, at the same time are of both scientific and industrial importance. However, catalysts of sufficient activity, selectivity and stability under application-relevant reaction conditions are not yet available. To date, many catalysts have been proposed for NH_3_-SCO and they can be classified into several groups, including noble metal-based catalysts, transition metal-based catalysts and noble/transition metal (bimetallic)-containing catalysts, etc. Particularly, the catalysts containing copper species are recognized as the most active and N_2_ selective among other transition metal-containing materials. Copper oxide species and their redox properties were found to determine their catalytic properties [4]. Recently, we published on noble metal-based catalysts (including Pt-, Pd-, Ag-, and Au-, Ru-based catalysts) [5] and Cu-containing zeolite-based catalysts (e.g., Cu-SSZ-13 commercialized in NH_3_-SCR-DeNO*_x_*) [6]. Thus, in the current mini-review, we focused on the application of transition metal oxides, excluding catalysts modified with noble metals or zeolite-based catalysts. However, the publications and results on the subject of transition metal-based catalysts increased recently, therefore, we found the mini-review timely. Particularly, we aim to compare hydrotalcite-based mixed metal oxides with other transition metal catalysts presented in the literature, and based on that propose an active, N_2_ selective, and stable catalytic system (either single one or as a component for hybrid catalyst) for NH_3_-SCO. The hybrid catalyst consists of an SCR catalyst for NO*_x_* control and catalyst with oxidation functionality for ammonia conversion. The different arrangements of the hybrid catalysts, i.e., dual-layer, inverse dual-layer, hybrid dual layer, dual mixed layers, etc., were so far tested in the literature (e.g., [7]). The combination of the two active components for NH_3_-SCR-DeNO*_x_* and NH_3_ oxidation to yield NO*_x_* arise from the internal selective catalytic reduction mechanism (i-SCR) [3,4]. However, despite research in this direction, a debate remains on the elementary reaction steps and the active sites in NH_3_-SCO. Moreover, for the i-SCR mechanism, the imide mechanism (with the formation of imide (-NH) and nitrosyl (-HNO) as intermediates), and the hydrazine mechanism (involving hydrazine (N_2_H_4_) as an intermediate) are reported as the main mechanisms for NH_3_-SCO. Recently, the N_2_^−^ mechanism (with adsorbed N_2_ anion regarded as the intermediate), was also proposed to explain the high reactivity of nano-size Al_2_O_3_-supported Ag species [8]. Contrary to the micro-size Al_2_O_3_ supported Ag species, the i-SCR mechanism was proposed, evidencing that the reaction mechanism depends on the applied catalytic systems. Another example can constitute Cu species deposited on Al_2_O_3_. Depending on the applied treatment (calcination *versus* dynamic construction), NH_3_-SCO over the catalysts can follow different routes. For example, the fast i-SCR mechanism characterized by the presence of consumable NO_2_ adsorbed species was proposed on CuO*_x_*-OH interfacial sites [9]. Still, compared to NH_3_-SCR-DeNO*_x_*, the reaction mechanisms of NH_3_-SCO are not frequently discussed in the literature. Hence, it is vital to study the reaction mechanisms over more materials thoroughly and then rationally design high-performing NH_3_-SCO catalyst with appropriate promotion strategies.

Although some review articles [3,4,10] have outlined the advances of transition-metal-based catalysts in NH_3_-SCO, only examples of such materials have been given. Thus, in the current mini-review, we thoroughly discuss the hydrotalcite-derived mixed metal oxides and other transition metal oxides applied in NH_3_-SCO, evidencing that for these materials their application as catalysts is quite relevant. Our objective was not to make a systematic review of the hydrotalcite-like compounds because several reviews have already been published, including preparation and physico-chemical characterization (e.g., [11,12,13]), catalytic applications (e.g., [14,15])—in particular nitrogen oxides removal [16,17]. Despite not being mentioned in the text, selective ammonia oxidation over (mixed) metal oxides into NO [18,19,20,21,22] or N_2_O [23,24] was reported, in the present work we concentrate only on the ammonia oxidation into N_2_ and H_2_O. Throughout the mini-review, we highlight the structure-activity/selectivity correlations and try to narrow the gap between research and industrial applications. We hope that based on these correlations, a knowledge-driven industrial catalyst design and its optimization becomes possible, which will allow keeping the NH_3_ emissions of diesel-powered vehicles at a very low level under various boundary conditions.

## 2. Hydrotalcite-Derived Mixed Metal Oxides

**Figure 2:**
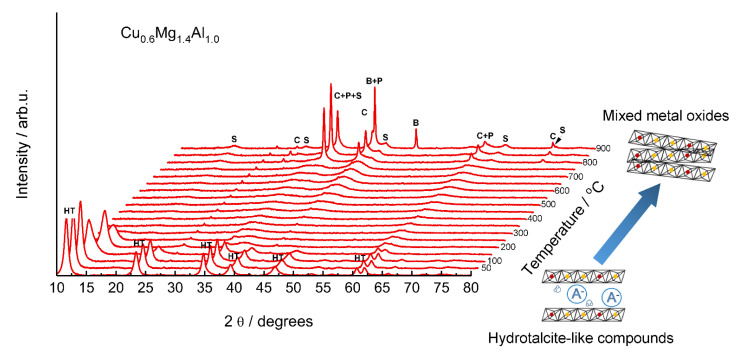
In situ XRD diffraction patterns of the Cu-Mg-Al hydrotalcite-like material recorded in oxidizing conditions. HT—hydrotalcite-like compounds, P—MgO (periclase), C—Cu_2_O (cuprite), S—MgAl_2_O_4_ (magnesium aluminate) and/or CuAl_2_O_4_ (copper aluminate), B—CuAlO_2_: Reprinted from [28] with permission from Springer.

Hydrotalcite-like compounds (HT), otherwise referred to as anionic clays or layered double hydroxides (LDHs) are described by the general formula [M(II)_1−*x*_M(III)*_x_*(OH)_2_]*^q^*^+^(A*^n^*^−^)*_q/n_*) mH_2_O, where M(II) and M(III) represent divalent and trivalent metal cations, respectively, A*^n^*^−^ represents interlayer anions of charge *n*^−^. Usually, the structure of the HT-like compounds is better visualized by analyzing the structure of brucite. Mg(OH)_2_ octahedra of Mg^2+^ coordinated with six OH^−^ share edges to form successive sheets, with the hydroxide ions located perpendicularly to the plane of the layers. The resulting sheets are stacked on top of each other and held together by hydrogen bonds. When Mg^2+^ ions are substituted by Al^3+^, a positive charge is created in the hydroxyl part of the layer. The positive charge is neutralized by the negative CO_3_^2−^ anions, which are located between the layers of brucite, along with H_2_O that is also present in the interlayer space (Figure 2) [25,26]. To obtain a pure hydrotalcite-like phase, the *x* in the general formula of the material should be in the range of 0.15 and 0.34 [25]. Coprecipitation is the most common method for the synthesis of hydrotalcite-like compounds.

The structure and physico-chemical properties of the hydrotalcite-like compounds are dependent on the kind and amount of metal ions present in the brucite-like layers, the type and position of anions and water in the interlayer region, and the type of stacking between the layers (i.e., rhombohedral (3R) *versus* hexagonal (2H)) [27]. It is possible to synthesize hydrotalcite-like compounds with more than two different metals (regarding different oxidation states, e.g., Li, Mg, Mn, Fe, Co, Al, Mn, Fe, Co, Ni, Cr, Ga) or anions (halides: Cl^−^, F^−^, I^−^, oxo-anions: CO_3_^2−^_,_ NO_3_^−^, SO_4_^2−^, BrO_3_^−^, organic acids: adipic, oxalic, sebacic or malonic acid, oxo and polyoxo-metallates: (PW_12_O_40_)^3−^, (PMo_12_O_40_)^3−^, chromate, dichromate, anionic complexes: ferro and ferricyanide, PdCl_4_^2−^, etc.). Consequently, the size of the interlayer region varies depending on the introduced anions. e.g., Table 1 lists the products obtained from the preparation of CuM(II)M(III)CO_3_ hydrotalcite-like compounds.

The hydrotalcite-like compounds are used as the precursors for the catalysts more often than as layered materials themselves. During the thermal treatment, the HT-like compounds transform first to an amorphous oxide and then, at higher temperatures, to crystalline mixed metal oxides (Figure 2). The hydrotalcite-derived mixed metal oxides are characterized by key features such as relatively high specific surface area, homogenous dispersion of active metal ions, non-stoichiometry, and high thermal stability, etc. [15].

**Figure 3:**
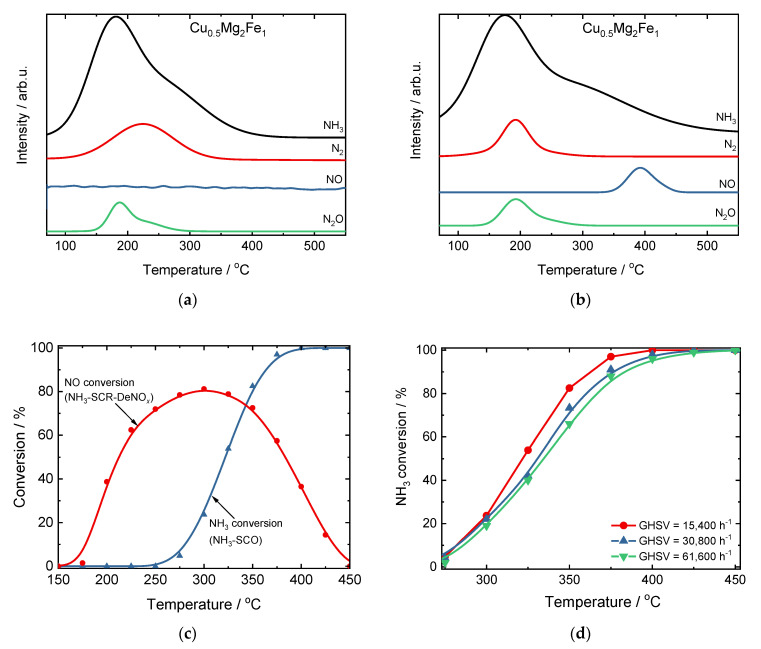
Results of temperature-programmed desorption of NH_3_ in (**a**) pure He or (**b**) 5 vol.% O_2_/He, adsorption: 70 °C, 1 vol.% NH_3_/He, (**c**) comparison of conversion of NH_3_ and NO, and (**d**) comparison of the space velocities (SV) over the CuFeAl hydrotalcite-derived mixed metal oxides. Reprinted from [31] with permission of Springer.

To the best of our knowledge, Trombetta et al. [29] reported for the first time the catalytic activity and N_2_ selectivity over the CuMgAl hydrotalcite-derived mixed metal oxides in NH_3_-SCO in 1997. They tested CuMgAl with *n*(Cu)/*n*(Mg)/*n*(Al) of 4.6–7.2/63.8–66.4/29 and found nearly full NH_3_ conversion at ca. 400–500 °C and N_2_ selectivity below 80%. Following such studies, Chmielarz et al. [30] studied the activity of hydrotalcite-derived mixed metal oxides (M(II, III)Mg(II)Al(III)) containing Ni, Fe, Cu or Co, and pointed out that both the kind and the number of metal ions introduced into hydrotalcite-like structure influenced the activity and selectivity in NH_3_-SCO. Among the investigated compositions, Cu-containing catalysts (CuMgAl; *n*(Cu)/*n*(Mg)/*n*(Al) = 5/66/29, 10/61/29, 20/51/29) were the most active in NH_3_-SCO, while the Fe-containing one (FeMgAl; *n*(Fe)/*n*(Mg)/*n*(Al) = 10/61/29) revealed enhanced N_2_ selectivity. Based on these results, the catalytic properties were further optimized by the combination of both metals, i.e., the introduction of copper and iron ions into the brucite-like structure. The CuMgFe mixed metal oxides with different compositions, such as *n*(Cu)/*n*(Mg)/*n*(Fe) = 0–1/2/1, mol.% (with the optimum composition guaranteeing enhanced NH_3_ conversion and N_2_ selectivity being *n*(Cu)/*n*(Mg)/*n*(Fe) = 0.5/2/1 [31]) or *n*(Cu)/*n*(Mg)/*n*(Fe) = 5–15/52–62/33 (with the optimum composition of *n*(Cu)/*n*(Mg)/*n*(Fe) = 12/55/33 [32]) were reported. The temperature-programmed studies, i.e., sorption of NH_3_ and desorption in He or O_2_/He, as well as NH_3_-SCR-DeNO*_x_* and NH_3_-SCO with different spaces velocities, revealed that the reaction over the CuMgFe hydrotalcite-derived mixed metal oxides proceeds according to the i-SCR mechanism and NH_3_ oxidation to NO is a rate-determining step (Figure 3) [31]. Thus, the modification of hydrotalcite-derived materials with noble metals (Pt, Pd, Rh) arose based on those studies [33].

**Figure 4:**
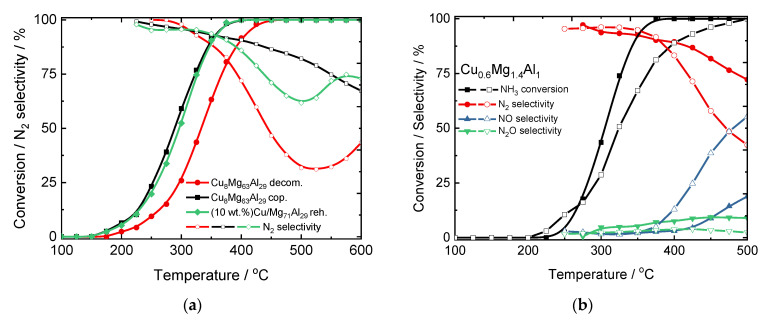
(**a**) Results of NH_3_-SCO over the CuMgAl mixed metal oxides. Reprinted from [34] with permission from Elsevier, and (**b**) results of NH_3_-SCO over the hydrotalcite-derived mixed metal oxides calcined at 600 and 900 °C. Reprinted from [28] with permission from Springer.

Copper loading at about 5–8 mol.% in the CuMgAl mixed metal oxides allowed reaching full NH_3_ conversion at 375–600 °C with N_2_ selectivity above 60% [34]. The increase in copper loading led to the formation of bulk-like copper oxide species. The N_2_ selectivity varied depending on the catalysts’ composition and the method used for the preparation of the hydrotalcite-like precursor. CuMgAl mixed metal oxides prepared via coprecipitation (cop.), and subsequent calcination of the hydrotalcite-like precursor revealed a significantly higher activity and N_2_ selectivity compared to the material with similar composition obtained via rehydration (reh.) of calcined Mg-Al hydrotalcite-like compounds or thermal decomposition (decom.) of nitrate precursors (Figure 4a). This effect was ascribed to the presence of the highly dispersed copper oxide species in hydrotalcite-derived mixed metal oxides. Additionally, the catalysts containing the same copper content, but with variations in the *n*(Mg)/*n*(Al) ratio presented similar catalytic activity [34,35]. Beyond the optimization of the kind and loading of metal species, the optimization of the calcination temperature is also relevant. The thermal treatment of hydrotalcite-like materials influences the composition of mixed metal oxides and their physico-chemical properties and thus, the activity and N_2_ selectivity in NH_3_-SCO [28,35]. Hydrotalcite-derived CuMgAl, CuZnAl, CuMgFe mixed metal oxides calcined at 900 °C revealed significantly lower activity compared to the materials calcined at 600 °C (Table 2, pos. 5, Figure 4b), which was ascribed to the different copper oxide phases and their redox properties. Enhanced activity at low temperatures together with a drop of N_2_ selectivity at higher temperatures were driven by the easily reduced copper oxides species. Otherwise, calcination temperature at ca. 800 °C led to the formation of the spinel phases, e.g., Cu_1−*x*_Mg*_x_*Al_2_O_4_ of lower reducibility, which caused higher N_2_ selectivity [35].

Regarding the use of other metals as dopants in Cu-containing mixed metal oxides, Jabłońska et al. [36] introduced Ag, Ce and Ga (*y* = 0–1 mol.%) to the CuMgAl mixed metal oxides (*n*(M)/*n*(Cu)/*n*(Mg)/*n*(Al) = *y*/5/66-*y*/29). The redox properties determined the catalytic properties for materials with the loading of *y* ≤ 0.25, while for the higher metal loading (*y* ≥ 0.25) the catalytic properties were driven mainly by the metal oxide phases. Górecka et al. [37] investigated hydrotalcite-derived (5 mol.%) CuMgAl mixed metal oxides, also impregnated with cerium (4 wt.%) over different feed compositions, i.e., NH_3_ and O_2_ (2 vol.% *versus* 20 vol.%). Higher O_2_ concentration enhanced NH_3_ conversion, while N_2_ selectivity dropped (the opposite effect was found for higher NH_3_ concentration in the feed). The effect of the enhanced NH_3_ activity over Cu-doped samples was ascribed to the synergetic effect of Ce-Cu redox pairs, which activated the lattice oxygen to react with NH*_x_* species towards the formation of N_2_ (Equation (5)). The oxygen vacancies were filled again with the surface oxygen as the cerium and copper species were oxidized (Equation (6), where ⎕ represents oxygen vacancies):

Ce^4+^ − O^2−^ − Cu^2+^ + NH*_x_* → Ce^3+^ − ⎕ − Cu^+^ + N_2_ + H_2_O (5)

Ce^3+^ − ⎕ − Cu^+^ + O_2_ → Ce^4+^ − O_2_ – Cu^2+^ (6)

However, from such studies, it was not clear why a rather high Ce loading was applied, since previous research showed that lower cerium loading (0.5 wt.% *versus* 3 wt.%) led to improved catalyst activity in NH_3_-SCO [38]. Nevertheless, even higher Ce loading (8.14 wt.%) was applied in further studies over the hydrotalcite-derived CuZnAl mixed metal oxides [39]. Overall, such systems revealed significantly lower N_2_ selectivity compared to the Ce/CuMgAl mixed metal oxides (Table 2, pos. 10). N_2_O was a minor by-product. Nevertheless, it is also worth mentioning that the Co-Mn-containing materials were reported to selectively oxidize NH_3_ to N_2_O (ca. 100% below 250 °C) [40].

Concluding, the above-mentioned examples show that the hydrotalcite-derived mixed metal oxides offer a large variety of possible modifications and tuning of their properties, which makes them suitable for NH_3_-SCO applications. Mainly Cu-containing hydrotalcite-derived mixed metal oxides were applied for NH_3_ oxidation to N_2_. Overall, the full NH_3_ conversion between 375–650 °C and N_2_ selectivity above 70% (based on the data gathered in Table 2, depending on the catalyst composition and preparation, reaction conditions, etc.), were achieved over the Cu-containing hydrotalcite-derived mixed metal oxides. Thus, further optimization of both chemical and phase compositions could lead to enhanced NH_3_ conversion and N_2_ selectivity below 350 °C. Still, intensive studies focused on the development of such catalytic systems in NH_3_-SCO are required under application-relevant reaction conditions (i.e., minor NH_3_ slip (O_2_ excess), up to 600–700 °C (in the cycle of diesel particulate filter regeneration) in the presence of H_2_O, CO*_x_* and/or SO*_x_*), including an investigation of the reaction mechanisms.

## 3. Other Metal Oxides

In the literature, various types of other metal oxides (still excluding noble metal-doped catalysts and zeolite-based materials) have been reported for NH_3_-SCO. The investigation of Co_3_O_4_, MnO_2_, CuO, Fe_2_O_3_ and V_2_O_5_ in NH_3_-SCO was reported in the early studies of Il’chenko and Golodets in 1975 [41,42]. The specific catalytic activities at 230 °C (*p*(NH_3_) = 0.1 atm, *p*(O_2_) = 0.9 atm) of the selected metal oxides decreased in the following sequence: Co_3_O_4_, MnO_2_ > CuO > NiO > Bi_2_O_3_ > Fe_2_O_3_ > V_2_O_5_ > TiO_2_ > ZnO > WO_3_. Among the transition metals, V_2_O_5_, MoO_3_ and WO_3_ exhibited nearly 100% N_2_ selectivity at 230 °C [43]. A similar activity order (MnO_2_ > Co_3_O_4_ > CuO > Fe_2_O_3_ ≈ V_2_O_5_ > NiO) was found by Hinokuma et al. [44] under 1 vol.% NH_3_, 0.75 vol.% O_2_, He balance. CuO reached higher N_2_ selectivity than other oxides [43,45].

**Figure 5:**
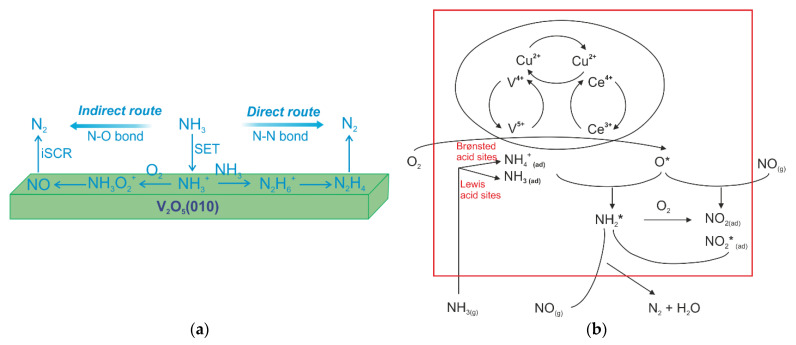
(**a**) Two competitive routes for NH_3_ oxidation over V_2_O_5_(010). Reprinted from [55] with permission from ACS Publications; (**b**) schematic representation of the mechanism of NH_3_-SCO over Cu-Ce/V_2_O_5_-WO_3_-TiO_2_. Reprinted from [53] with permission from Elsevier.

NH_3_-SCO (as a side process) has been frequently studied with NH_3_-SCR-DeNO*_x_*, thus, the following studies focused on the supported V-containing catalysts, e.g., V_2_O_5_/TiO_2_ [46,47] V_2_O_5_/TiO_2_-SiO_2_ [48], V_2_O_5_-WO_3_/TiO_2_ [46,49], V_2_O_5_-WO_3_/ZrO_2_ [50], etc. E.g., Ueshima et al. [51] examined different supports for V_2_O_5_-WO_3_ and found the decreasing order of catalytic activity in terms of support as follows: TiO_2_-SiO_2_ (binary oxide) > TiO_2_ (anatase) > TiO_2_ (rutile) > SiO_2_. Contrary to such studies, V_2_O_5_ supported on the rutile form of TiO_2_ led to a more active and N_2_ selective (below 400 °C) catalyst [47]. In another study, V_2_O_5_/MgO was the least active among vanadium oxide supported on TiO_2_, SiO_2_ or MgO [52]. Furthermore, the commercial V_2_O_5_-WO_3_-TiO_2_ catalyst was modified with Cu (1 wt.%) and Ce (1–10 wt.%) species. Overall, the catalyst with the composition of (1.02 wt.%)Cu-(4.79 wt.%)Ce/V_2_O_5_-WO_3_-TiO_2_ showed enhanced activity at 300 °C, while its modification with Ce enhanced H_2_O and sulfur resistance [53]. The V_2_O_5_-WO_3_-TiO_2_ catalyst modified with Cu or Fe (1.0 wt.%) ions revealed the highest activity and N_2_ selectivity above 350 °C (among other materials modified with Mn or Co species) [54]. Furthermore, K_2_O was also suggested (but not experimentally tested) as an effective promoter for the V_2_O_5_/TiO_2_ and V_2_O_5_-WO_3_/TiO_2_ catalysts for NH_3_-SCO at 500 °C. Regarding, the reaction mechanisms in NH_3_-SCO, Yuan et al. [55] performed density functional theory (DFT) calculations in conjunction with cluster models on the V_2_O_5_ surfaces. According to such mechanisms (Figure 5a), NH_3_^+^ appears as the initial intermediate from the activated NH_3_ which transfers an electron to the metal oxide surfaces. Further routes, depending on the availability of the O_2_ species, can arise, i.e., the *direct route* appears in the case of limited O_2_ or its absence. Consequently, formed N_2_H_4_ was oxidized to N_2_ on V=O sites. In the presence of O_2_, NH_3_O_2_^+^ complex was formed, which further decomposes to NO (followed by NH_3_-SCR-DeNO*_x_*) with the N_2_ formation. Such an *indirect route* (i.e., i-SCR mechanism) was reported also over the V-based catalysts [53,54] (e.g., Figure 5b). Recently, Liu et al. [56] identified based on the first-principle calculation method of DFT the adsorption sites of NH_3_ on V_2_O_5_(001).

Carley et al. [57] revealed the structural characteristics of imide strings formed when a Cu(110) surface was exposed to the NH_3_-O_2_ mixture (30 vol.%–1 vol.%, 52 °C, 10^−8^ mbar —UHV investigations). Contrary to that, no imide species were found on the surface of polycrystalline copper in NH_3_-SCO at 1.2 mbar [58]. CuO was found to selectively oxidize NH_3_ to N_2_O, while Cu_2_O to N_2_, respectively [58,59]. Hirabayashi and Ichihashi [60] investigated reactions of copper oxide cluster cations, Cu*_n_*O*_m_*^+^ (*n* = 3–7, *m* ≤ 5) with NH_3_ at near thermal energies using a guided ion beam tandem mass spectrometer. Depending on the applied clusters, H_2_O, O_2_ or -HNO were released, while the release of N_2_ was observed in the multiple-collision reactions of Cu_5_O_3_^+^ and Cu_7_O_4_^+^ clusters. Nevertheless, it is recognized that supported copper species (e.g., Cu/Al_2_O_3_ [61,62], CuO/carbon nanotubes [63]) or the combination of CuO and other transition or rare earth metal oxides, e.g., Fe_2_O_3_, CeO_2_, La_2_O_3_, CuCr_2_O_4_ and CuCrO_2_ or La_2_Ce_2_O_7_ (nonporous pyrochlore structure—A_2_B_2_O_7_) (e.g., [61,62,64,65,66,67]) result in catalysts with enhanced activity and N_2_ selectivity in NH_3_-SCO. E.g., Gang et al. [61,62] and afterward other authors [68,69,70,71] have proved the high catalytic activity of copper species deposited on γ-Al_2_O_3_. They claimed that copper species dispersion becomes poorer on Al_2_O_3_ at metal loading higher than ca. 10 wt.% (among 5–15 wt.%) [62]. Furthermore, many studies about similar catalytic systems concur on ca. 10 wt.% as an optimum loading of copper species [4]. The full NH_3_ conversion of this group appears at 350–500 °C with N_2_ selectivity above 75% (Table 2) depending on the preparation methods (e.g., copper precursors [70,72], treatment strategies [9]), and reaction conditions (e.g., fuel-lean/rich conditions [71,73,74]). Based on the studies of Lenihan and Curtin [69], the stability of Cu/Al_2_O_3_ through the dry, wet and subsequently dry conditions was proved. Moreover, the activity of CuO/Al_2_O_3_ catalyst was further enhanced by its dopping with PbO, NiO, CoO or SnO (with ≤ 1 wt.% loading of dopant) [68]. Not only has γ-Al_2_O_3_ been applied as the catalyst support, η-Al_2_O_3_ has also been employed [75]. Still, the full NH_3_ conversion appeared around 550 °C over (1–2 wt.%)Cu/η-Al_2_O_3_. The catalytic properties of Cu/Al_2_O_3_ were further increased via its modification with Li_2_O and CeO*_x_*. Above 250 °C the materials were only N_2_ selective [76]. In addition, increasing the *c*(O_2_)/*c*(NH_3_) ratio enhanced the conversion. Recently, Machida et al. [77] investigated several nanometer-thick transition metal (mainly noble metals but also Cu or Co) overlayers formed on a Fe-Cr-Al metal (SUS) foil by pulsed cathodic arc-plasma deposition. The activity of the Co- and Cu-based materials decreased significantly in the presence of 10 vol.% H_2_O (while those of the catalysts containing Pt and Ir remained nearly unchanged, i.e., preserved the active metallic surface during NH_3_-SCO in the presence of O_2_/H_2_O).

Other than Cu, other metal species such as Ni, Mn, Fe, Co, Mg and Zn were supported on Al_2_O_3_ [78]. For the (10 wt.%)Ni-containing catalysts, the activity was found to decrease in the order of γ-Al_2_O_3_, ZrO_2_, MgO > SiO_2_ > TiO_2_ >ZSM-5, with full NH_3_ conversion being reached at 550 °C for Ni/Al_2_O_3_ (possibly due to presence of NiAl_2_O_4_). The catalytic properties of the samples loaded above 10 wt.% tend to become similar to that expected for pure NiO. Furthermore, Mn/Al_2_O_3_ and Fe/Al_2_O_3_ were proved to be more active (full NH_3_ conversion at 300–500 °C with N_2_ selectivity > 70%) than Ni/Al_2_O_3_ (550 °C) for NH_3_ oxidation, possibly because of their enhanced redox properties. In addition, several groups have investigated Ni-based catalysts above 500 °C (e.g., prepared via microemulsion) [79,80].

**Figure 6:**
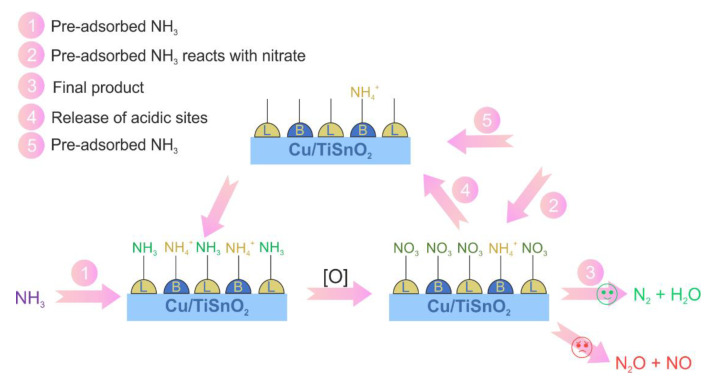
Reaction mechanism of NH_3_-SCO over Cu/TiSnO_2_. Reprinted from [85] with permission of Elsevier.

He et al. [65] demonstrated that TiO_2_ is a more suitable support (due to the higher oxygen mobility and lower oxygen bonding strength) than Al_2_O_3_ for copper-based catalysts, which was represented by the enhanced catalytic properties of Cu/TiO_2_ compared to Cu/Al_2_O_3_. Contrary to that, for the Cu-Mn species, deposition on Al_2_O_3_ (compared to TiO_2_) guaranteed enhanced activity [81]. In the case of Cu/TiO_2_, NH_3_ conversion was reported to depend on the Cu species loading, e.g., for (10 wt.%)Cu/TiO_2_ full conversion occurs at about 250 °C with 95% N_2_ selectivity [65], while for (1 wt.%)Cu/TiO_2_—425–500 °C with < 60% N_2_ selectivity was reported [82]. Duan et al. [83] investigated (10 wt.%) V, Cr, Zn and Mo supported on TiO_2_. Comparatively tested Cu/TiO_2_ and Cr/TiO_2_ revealed lower NO and NO_2_ selectivity over chromium-containing catalysts. The applied O_2_ content in the feed gas ranging from 0.5 vol.% to 5 vol.% revealed similar NH_3_ conversion (with an exception below 150 °C where NH_3_ conversion was higher in the presence of 0.5 vol.% O_2_). Moreover, NO selectivity was not affected by the different O_2_ content during NH_3_-SCO. TiO_2_ anatase is the most common catalyst support, while the catalytic properties are affected by the properties of the support (i.e., anatase *versus* rutile) [84]. NH_3_-SCO over Cu/TiSnO_2_ and Cu/TiO_2_ (10.5–11.8 wt.% of Cu) was reported to follow the i-SCR and imide (-NH) mechanisms, respectively [85]. For the i-SCR mechanism (Figure 6), NH_3_ was first adsorbed on both Lewis and Brønsted acid sites. After that, it reacted with surface-active oxygen species to form nitrate species (intermediates in NH_3_-SCO), which finally reacted with the remaining NH_3_ with the formation of N_2_ and H_2_O. Gaseous NH_3_ recombined with the released acid sites to participate in the next cycles.

The bands assigned to nitrate species were also found using in situ DRIFTS in the spectra of a series of CuO-Fe_2_O_3_ catalysts (with an optimum at *n*(Cu):*n*(Fe) molar ratio of 5:5 with full NH_3_ conversion > 250 °C) recorded at 250 and 350 °C [86]. The authors proposed the molecular steps of the i-SCR mechanism, in which the nitrosyl (-HNO) species were formed via a reaction between adsorbed NH_3−*x*_ species with atomic oxygen (Equations (7)–(10)). Then, the -HNO was oxidized by oxygen atoms from O_2_ to form NO species (Equations (11) and (12)). Additionally, the -NH species interacted with O_2_ to form NO. Meanwhile, the in situ-formed NO could react with -NH*_x_* to form N_2_ or N_2_O (Equations (13) and (14)).

NH_3_ → NH_2_ + H (7)

NH_2_ → NH + H (8)

NH + O → HNO(9)

O_2_ → 2O (10)

HNO + O → NO + OH(11)

NH + O_2_ → NO + OH (12)

NH_2_ + NO → N_2_ + H_2_O (13)

NH + NO → N_2_O + H (14)

H + OH → H_2_O (15)

To reveal the impact of calcination temperature on the catalytic properties of the CuO-Fe_2_O_3_ catalysts (at *n*(Cu)/*n*(Fe) molar ratio of 1/1), the materials were calcined between 400 and 700 °C [87]. Among them, CuO-Fe_2_O_3_ calcined at 500 °C revealed full NH_3_ conversion at ca. 225 °C, i.e., at about 25 °C lower than for the material calcined at 400 °C. The increase in the calcination temperature (up to 600–700 °C) resulted in a decrease in the activity in NH_3_-SCO, while selectivity was not affected. The simultaneous addition of H_2_O and SO_2_ to the feed gas led to a drop in activity and N_2_ selectivity. Regarding the application of the Cu-Fe-containing spinel, Yue et al. [88] found that for the mesoporous CuFe_2_O_4_—prepared with KIT-6 as the hard template, NH_3_ was nearly completely consumed at 300 °C while the N_2_ selectivity dropped below 90% up to 600 °C. CuMoO_4_, CoMoO_4_ or FeMoO_4_ were significantly less active in NH_3_-SCO [45]. For CuMoO_4_, activity and N_2_ selectivity were completely inhibited by water vapor (10 vol.%). Beyond fully synthesized materials, natural vermiculite and phlogopite [89,90,91] or attapulgite [92] modified with Cu or Fe species are also active and N_2_ selective catalysts for NH_3_-SCO (Table 2, pos. 42–46).

**Figure 7:**
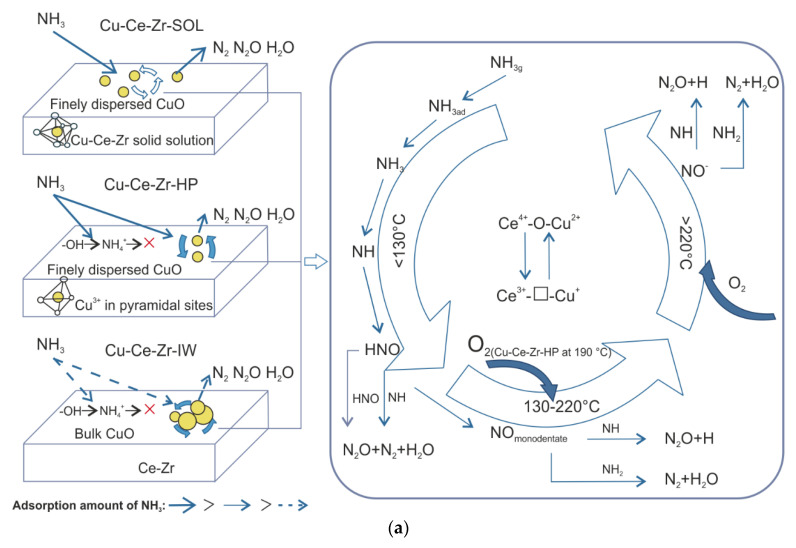

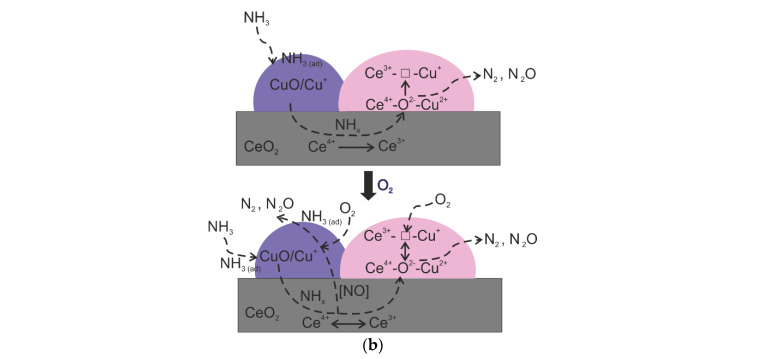
(**a**) Relationship of activity-adsorption-structure and reaction pathway over Cu-Ce-Zr prepared via different synthesis routes; SOL—citric acid sol-gel method; HP—homogeneous precipitation, IW— incipient wetness impregnation. Reprinted from [98] with permission from Elsevier; (**b**) NH_3_-SCO oxidation over CuO-CeO_2_. Reprinted from [100] with permission from Elsevier.

Similar to pure CuO and NiO, for CeO_2_ the catalytic activity was also poor [93,94]. Despite this, the (10 wt.%)Ce/TiO_2_ catalyst (calcined at 400–500 °C) revealed enhanced activity in NH_3_-SCO between 300–350 °C but did not reach full NH_3_ conversion [94]. Furthermore, the catalytic activity increased from 50 to 90% at 300 °C for (10 wt.%)Ce/TiO_2_ after its modification with vanadium (2 wt.%) [95]. This effect was assigned to the dispersion of Ce^4+^ species on TiO_2_. The V/Ce/V/TiO_2_ catalyst showed resistance to SO_2_ poisoning due to the reduced formation of the NH_4_HSO_4_ species. The Ce-containing mixed metal oxides constitute a representative group of catalysts for NH_3_-SCO. E.g., Wang et al. [93] investigated a series of Ce_1−*x*_Zr*_x_*O_2_ (0.2 ≤ *x* ≤ 0.8) mixed oxide catalysts, among which particularly Ce_0.4_Zr_0.6_O_2_ reached the total NH_3_ oxidation of about 360 °C (N_2_ selectivity > 90%). Ce_0.4_Zr_0.6_O_2_ was also subjected to further modifications with Ru species [96]. Cu-Ce-Zr catalyst prepared by a citric acid sol-gel method exhibited the highest activity among other materials (prepared via the homogenous precipitation and incipient wetness impregnation methods) achieving full NH_3_ conversion at 230 °C with > 90% N_2_ selectivity [97]. These results were attributed to the finely dispersed CuO, the Cu-Ce-Zr solid solution and the monomeric Cu^2+^ ions in octahedral sites (in contrast to monomeric Cu^2+^ in the square-planar pyramidal sites). Moreover, the adsorbed oxygen species were more active than the bulk lattice oxygen species in NH_3_-SCO. The co-presence of SO_2_ and H_2_O or CO_2_ in the feed resulted in the NH_3_ conversion decreasing to 92 and 81%, respectively. NH_3_-SCO over the catalysts prepared via different techniques followed the i-SCR mechanism (with the -NH*_x_* and -HNO intermediates, Figure 7a) [98].

Lou et al. [99] have reported nearly complete NH_3_ conversion at temperatures as high as 400 °C with an overall N_2_ selectivity varying from 19 to 82% over Cu-Ce mixed oxides prepared by coprecipitation with an optimum at *n*(Cu)/*n*(Ce) = 6/4 (among 6–9/1–4). Afterward, the CuO-CeO_2_ catalysts prepared by a surfactant-templated method exhibited full NH_3_ conversion below 300 °C with more than 90% N_2_ selectivity [100]. However, the thermal resistance of CuO-CeO_2_ mixed oxides needs to be further enhanced. The finely dispersed CuO species as well as a strong synergetic interaction between the copper oxide species and cerium oxides significantly decreased the operation temperature. Thus, activated ammonia reacted with lattice oxygen in the Cu-O-Ce solid solution generating N_2_ and H_2_O, while gaseous O_2_ regenerated the oxygen vacancies in the Cu-O-Ce solid solution to maintain Ce^4+^/Ce^3+^ redox couple (Figure 7b). NH_3_ was oxidized over CeO_2_ to NO, which in the next step reacted with NH*_x_* forming N_2_ over CuO (according to the i-SCR mechanism). CuO/La_2_O_3_ (*n*(Cu)/*n*(La) = 6–9/1–4 with an optimum at 8/2) showed a significantly lower activity and N_2_ selectivity of 93 and 53% at 400 °C, respectively [64], compared to CuO-CeO_2_ (e.g., 98–99% NH_3_ conversion with 85–86% N_2_ selectivity at 400 °C for *n*(Cu)/*n*(Ce) = 6/4) [101,102].

Mn-based catalysts have been demonstrated to be active in NH_3_-SCO. E.g., natural manganese ore (NMO, consisting of manganese oxides and small amounts of Fe_2_O_3_, CaO, MgO, SiO_2_, Al_2_O_3_) was recognized as a low-cost catalyst possessing similar activity (ca. 50 % NH_3_ conversion) to that of MnO_2_ below 150 °C. Above 150 °C, Mn_2_O_3_ was the most active one. Across the studied temperatures between 50–250 °C, N_2_ selectivity decreased as follows: NMO > MnO_2_ > Mn_2_O_3_ [103]. Mn_2_O_3_ supported on TiO_2_ (anatase containing 1.15 wt.% sulfate) revealed significantly lower activity at 277–307 °C than the supported Cu-Mn mixed oxides (*n*(Cu)/*n*(Mn) = 20–80/20–80) [104]. It was found that the most active catalyst (with *n*(Cu)/*n*(Mn) = 20/80) contained a Cu_1+*x*_Mn_2−*x*_O_4_ crystalline spinel phase and X-ray amorphous Mn^2+^-containing species. Unfortunately, the selectivity was not reported. The activity of Cu-Mn/TiO_2_ was further enhanced via its modification with Ce or La species [105]. Ce-Cu-Mn/TiO_2_ prepared through the sol-gel method was the most active among the catalysts prepared via impregnation or coprecipitation and reached complete NH_3_ conversion at ca. 200 °C, however with 96% NO selectivity. Song et al. [106] investigated a series of MnO*_x_*(*z*)-TiO_2_ [106] (*z* = 0.1–0.3) prepared by the sol-gel method. The optimum MnO*_x_*(0.25)-TiO_2_ showed nearly full NH_3_ conversion around 200 °C with N_2_ selectivity of more than 60% up to 350 °C. NO was formed only above 250 °C. Based on the in situ DRIFTS studies, they claimed that N_2_ appeared as a product of the reaction between -HNO and -NH species. N_2_O was formed from the combination of two -HNO species at low temperatures, as well as from the reaction between adsorbed NH_3_ and nitrite/nitrate species at high temperatures. Additionally, the i-SCR mechanism was proposed over Fe_2_O_3_-Al_2_O_3_, Fe_2_O_3_-TiO_2_, Fe_2_O_3_-ZrO_2_ and Fe_2_O_3_-SiO_2_ prepared by the sol-gel method [107]. The materials prepared from iron sulfate led to a higher N_2_ selectivity than those prepared from nitrate. The higher N_2_ selectivity was reported earlier for CuO/TiO_2_ prepared from CuSO_4_ compared to the corresponding catalyst prepared from Cu(NO_3_)_2_ [52], which is also valid for the pre-sulfated samples [68].

**Figure 8:**
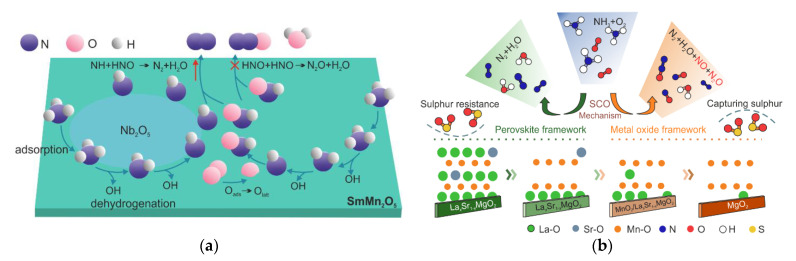
Schematic representation of (**a**) Nb_2_O_5_ modification to enhance the N_2_ selectivity of SmMn_2_O_5_. Reprinted from [108] with permission from Elsevier; (**b**) NH_3_-SCO mechanism over modified La*_x_*Sr_1-*x*_MnO_3_ perovskite. Reprinted from [110] with permission from ACS Publications.

Chen et al. [108] developed a series of mullite-based *A*Mn_2_O_5_ (*A* = Sm, Y, Gd) catalysts, among which SmMn_2_O_5_ achieved complete NH_3_ conversion at 175–250 °C (albeit with a rather low N_2_ selectivity of barely more than 45%). The imide mechanism was reported for NH_3_-SCO over SmMn_2_O_5._ Furthermore, its modification with niobium oxide (5 wt.%)Nb_2_O_5_/SmMn_2_O_5_ stood out with N_2_ selectivity > 60%. The niobium oxide was supposed to enhance the catalyst surface attraction to the N atom lone-pair electron. Consequently, the reaction between the increased amount of -NH and -HNO species towards the formation of N_2_ was favored (Figure 8a). In the other approach, SmMn_2_O_5_ was mixed with Cu-SAPO-34 [109]. Still, N_2_ selectivity varied between 20–60% in the range of 150–400 °C. For the mixed catalysts, the i-SCR mechanism was proposed (NH_3_ oxidation to NO*_x_* over mullite catalyst), which stays contrary to the above-mentioned studies. The i-SCR mechanism was also proposed over the La*_x_*Sr_1-*x*_MnO_3_ perovskite-based catalysts post-modified with a 3 M solution of HNO_3_ (0.1–72 h, Figure 8b) [110]. As the treatment time increased, the perovskite phase changed from a mixture of perovskite and MnO_2_ (10 h treatment) to pure MnO_2_ (72 h treatment). Additionally, the materials subjected to a 72 h treatment were the most active, albeit selective to NO and N_2_O, as well as poorly resistant to sulfur species.

Concluding, several transition metal-based catalysts were proposed for NH_3_ oxidation to N_2_. Among them, mainly Cu/Al_2_O_3_, Cu-Ce-Zr, CuO-Fe_2_O_3_ or CuO-CeO_2_ were the most frequently studied materials (according to data gathered in Table 2). In the case of Cu/Al_2_O_3_, the optimal loading of 10 wt.% Cu guarantees an enhanced NH_3_ conversion and N_2_ selectivity (full NH_3_ conversion at 350–500 °C with N_2_ selectivity above 75%). For the other mentioned materials, i.e., Fe- or Ce-containing systems, the catalytic studies were carried out only in a narrow temperature range. The complete NH_3_ conversion was achieved between 225/250–300 °C with N_2_ selectivity above 80% (Table 2). Further attention should be given to the stability under conditions simulating real exhaust from diesel engines up to 600–700 °C of the mesoporous CuFe_2_O_4_ spinel.

**Table 2:** Comparison of complete NH_3_ conversion and N_2_ selectivity in the same temperature range over hydrotalcite-derived mixed metal oxides and other transition metal-based catalysts reported in the literature (related data are marked with asterisks).

Pos.	Sample	Preparation	Reaction Conditions	Operation Temperature for Achieving 100% NH_3_ Conversion/°C	N_2_ Selectivity/%	Refs.
Hydrotalcite-derived mixed metal oxides						
1	CuMgAl *n*(Cu)/*n*(Mg)/*n*(Al) = 4.6/66.4/29, mol.%	Coprecipitation, calcination, 650 °C, air, 14 h	0.5 vol.% NH_3_, 1.75 vol.% O_2_, He balance, GHSV 10,000–12,000 h^−1^	500	>80	[29]
2	CuMgAl *n*(Cu)/*n*(Mg)/*n*(Al) = 5/66/29, mol.%	Coprecipitation, calcination, 600 °C, air, 16 h	0.5 vol.% NH_3_, 2.5 vol.% O_2_, He balance, GHSV 30,000 h^−1^	400–650	>80	[30]
3	CuMgFe *n*(Cu)/*n*(Mg)/*n*(Fe) = 0.5/2/1, mol.%	Coprecipitation, calcination, 600 °C, air, 12 h	0.5 vol.% NH_3_, 2.5 vol.% O_2_, He balance, GHSV 15,400 h^−1^	400–450	>70	[31]
4	CuMgAl *n*(Cu)/*n*(Mg)/*n*(Al) = 8/63/29, mol.%	Coprecipitation, calcination, 600 °C, air, 6 h	0.5 vol.% NH_3_, 2.5 vol.% O_2_, Ar balance, WHSV 24,000 mL h^−1^ g^−1^ * 0.5 vol.% NH_3_, 2.5 vol.% O_2_, N_2_ balance, WHSV 137,000–140,000 mL h^−1^ g^−1^ ** 0.5 vol.% NH_3_, 2.5 vol.% O_2_, 10 vol.% CO_2_, 5 vol.% H_2_O, N_2_ balance, WHSV 137,000–140,000 mL h^−1^ g^−1^	400–600 * 450–600 ** 600	>60 * >60 ** >55	[34] *^,^** [111]
5	CuMgAl *n*(Cu)/*n*(Mg)/*n*(Al) = 0.6/1.4/1.0, mol.%	Coprecipitation, calcination, 600 °C, *900 °C, air, 12 h	0.5 vol.% NH_3_, 2.5 vol.% O_2_, He balance, WHSV 24,000 mL h^−1^ g^−1^	375–500 * 500	>70 * >40	[28]
6	CuMgAl *n*(Cu)/*n*(Mg)/*n*(Al) = 5/62/33, mol.%	Coprecipitation, calcination, 600 °C, 800 °C *, air, 12 h	0.5 vol.% NH_3_, 2.5 vol.% O_2_, He balance, WHSV 24,000 mL h^−1^ g^−1^	475–500 * 475–500	>60 * >85	[35]
7	GaCuMgAl * CeCuMgAl *n*(Ga/Ce)/*n*(Cu)/*n*(Mg)/*n*(Al) = 0.25/5/65.75/29	Coprecipitation, calcination, 600 °C, air, 6 h	0.5 vol.% NH_3_, 2.5 vol.% O_2_, Ar balance, WHSV 24,000 mL h^−1^ g^−1^	375–500 * 375–500	>80 * >50	[36]
8	CuMgAl *n*(Cu)/*n*(Mg)/*n*(Al) = 10–15/52–57/33, mol.% * (4.1 wt.%)CeCuMgAl *n*(Cu)/*n*(Mg)/*n*(Al) = 5/62/33, mol.%	Coprecipitation, calcination, 800 °C, air, 9 h * Impregnation, calcination, 800 C, air, 9 h	0.035 vol.% NH_3_, 20 vol.% O_2_, N_2_ balance, WHSV 30,000 mL h^−1^ g^−1^	350 * 350	<20 * <70	[37]
9	CuMgAl *n*(Cu)/*n*(Mg)/*n*(Al) = 5/62/33, mol.% * (3 wt.%)CeCuMgAl ** (0.5 wt.% Ce)CuMgAl	Coprecipitation, calcination, 600 °C, air, 12 h, *^,^** Impregnation, calcination, air, 600 °C, 12 h	0.5 vol.% NH_3_, 2.5 vol.% O_2_, He balance, WHSV 24,000 mL h^−1^ g^−1^	500–600 * 500–600 ** 450–600	>60 * >75 ** >55	[38]
10	CuZnAl *n*(Cu)/*n*(Zn)/*n*(Al) = 10–15/52/33, mol.% * (8.14 wt.%)CeCuMgAl		0.035 vol.% NH_3_, 20 vol.% O_2_, N_2_ balance, WHSV 30,000 mL h^−1^ g^−1^	350 * 350	<30 * <40	[39]
11	CoMnAl *n*(Co)/*n*(Mn)/*n*(Al) = 4/1/1	Coprecipitation, calcination, 500 °C, air, 4 h; * Mechanochemical method, calcination, 500 °C, air, 4 h	0.5 vol.% NH_3_, 2.5 vol.% O_2_, He balance, WHSV 24,000 mL h^−1^ g^−1^	250–500 * 250–500	>40 * >45	[40]
Other metal oxides						
12	CuO/monolith	Precursors calcination on the monolith, 600 °C, air, 6 h	0.05 vol.% NH_3_, 3 vol.% O_2_, N_2_ balance, GHSV 40,000 h^−1^	450–550	67–85	[45]
13	(10 wt.%)V/TiO_2_	Impregnation, calcination, 550 °C, air, 6 h	0.05 vol.% NH_3_, 2.5 vol.% O_2_, N_2_ balance, GHSV 35,385 h^−1^	225–300	-	[83]
14	(10 wt.%)Cu/TiO_2_			200–300	-	
15	(10 wt.%)Cu/TiO_2_	Impregnation, rotary evaporator, calcination, 450 °C, air, 3 h	0.04 vol.% NH_3_, 10 vol.% O_2_, He balance, GHSV 50,000 h^−1^ * 0.04 vol.% NH_3_, 10 vol.% O_2_, 3 vol.% H_2_O, He balance, GHSV 50,000 h^−1^	250–300 * 350–375	>95 * >95	[65]
16	(10 wt.%)Cu/Al_2_O_3_			400	>95	
17	(10–15 wt.%)Cu/Al_2_O_3_	Impregnation, calcination, 600 °C, air, 24 h	1.14 vol.% NH_3_, 8.21 vol.% O_2_, He balance, WHSV 2 240 mL h^−1^ g^−1^	350	>90	[61]
18	(10 wt.%)Cu/Al_2_O_3_	Impregnation, calcination, 600 °C, air, 24 h	1.14 vol.% NH_3_, 8.21 vol.% O_2_, He balance, WHSV 2240 mL h^−1^ g^−1^ * 1.14 vol.% NH_3_, 8.21 vol.% O_2_, He balance, WHSV 2240 mL h^−1^ g^−1^	350 * 350	94 * 95	[62]
19	(10 wt.%)Cu/Al_2_O_3_	Impregnation, calcination, 600 °C, air, 3 h, * Cu(CH_3_COO)_2_ as precursor ** Cu(NO_3_)_2_ as precursor	0.1 vol.% NH_3_, 10 vol.% O_2_, He balance, GHSV 50,000 h^−1^	* 350–400 ** 375–400	* >85 ** >95	[70]
20	(10 wt.%)Cu/Al_2_O_3_	Impregnation, calcination, 600 °C, air, 6 h	0.5 vol.% NH_3_, 2.5 vol.% O_2_, N_2_ balance, WHSV 137,000–140,000 mL h^−1^ g^−1^ * 0.5 vol.% NH_3_, 2.5 vol.% O_2_, 10 vol.% CO_2_, 5 vol.% H_2_O, N_2_ balance, WHSV 137,000–140,000 mL h^−1^ g^−1^	450–600 * 600	>60 * >50	[111]
21	(10 wt.%)Cu/Al_2_O_3_	Impregnation, calcination, 600 °C, air, 12 h	0.5 vol.% NH_3_, 2.5 vol.% O_2_ Ar balance, WHSV 24,000 mL h^−1^ g^−1^	425–500	>75	[112]
22	(10 wt.%)Cu/Al_2_O_3_ * (10 wt.%)Cu/Al_2_O_3_	Imprgnation, rotary evaporation, calcination, 500 °C, air, 2 h * Impregnation, rotary evaporation, 500 °C, H_2_/N_2_, 2 h; 0.05 vol.% NH_3_, 5 vol.% O_2_, N_2_ balance	0.05 vol.% NH_3_, 5 vol.% O_2_ N_2_ balance, GHSV 60,000 h^−1^	330 * 300–330	not shown * not shown	[9]
23	(1.3 wt.%)Cu/Al_2_O_3_ * (1 wt.%)Cu/CeO*_x_*/Li_2_O/Al_2_O_3_	Impregnation, calcination, 350 °C, air, time not given; homogenous deposition precipitation, H_2_ reduction, 400 °C, 2 h	2 vol.% NH_3_, 2 vol.% O_2_, Ar balance, GHSV 2500 h^−1^	400 * 325–400	100 * 100	[76]
24	(3.4 wt.%)Cu/Al_2_O_3_	Impregnation, calcination, 450 °C, air, 5 h	0.54 vol.% NH_3_, 8 vol.% O_2_, He balance, WHSV 240 mL h^−1^ g^−1^	400–450	not shown	[69]
25	(20 wt.%)Cu/Al_2_O_3_/monolith	Impregnation, calcination, 800 °C, air, 4 h	0.04 vol.% NH_3_, 8.2 vol.% O_2_, 1.3 vol.% CH_4_, 3.9 vol.% CO_2_, 4.1 vol.% CO, 2.9 vol.% H_2_, GHSV 100,000 h^−1^	400–500	0	[71]
26	(1 wt.%)PbO-(4.3 wt.%)Cu/Al_2_O_3_	Impregnation, calcination, 450 °C, air, time not shown	0.54 vol.% NH_3_, 8 vol.% O_2_, He balance, WHSV 800 mL h^−1^ g^−1^	325	95	[68]
27	(1–2 wt.%)Cu/η-Al_2_O_3_	Impregnation, Rotary evaporator, calcination, 500 °C, air, 10 h; pre-treatment conditions: 20 vol.% O_2_/He, 550 °C, 1 h	0.1 vol.% NH_3_, 8 vol.% O_2_, 3.5 vol.% H_2_O, He balance, WHSV 250,000 mL h^−1^ g^−1^	550	not shown	[75]
28	CuO/CNTs (carbon nanotubes, 9.85 wt.% Cu)	Impregnation, ultrasonic treatmnet, evaporation, 350 °C, He, 3 h	0.1 vol.% NH_3_, 2 vol.% O_2_, He balance, WHSV 60,000 mL h^−1^ g^−1^	189–250	>98	[63]
29	Cu/graphene (2.57–3.42 wt.%)	Impregnation, ultrasonic treatment, 400 °C, N_2_, 3 h, * Cu(CH_3_COO)_2_ H_2_O as precursor ** Cu(NO_3_)_2_·H_2_O as precursor	0.05 vol.% NH_3_, 1 vol.% O_2_, N_2_ balance, GHSV 35,000 h^−1^	* 300 ** 250–300	* >80 ** >80	[72]
30	(5 wt.%)Ni/Al_2_O_3_	Impregnation, calcination, 800 °C, air, 8 h	0.1 vol.% NH_3_, 18 vol.% O_2_, N_2_ balance, GHSV 61,000 h^−1^	550–800	>55	[78]
31	(5 wt.%)Mn/Al_2_O_3_			300–800	>55	
32	(10.5 wt.%)CuO/TiSnO_2_	Impregnation, calcination, 450 °C, air, 4 h	0.05 vol.% NH_3_, 3 vol.% O_2_, N_2_ balance, WHSV 60,000 mL h^−1^ g^−1^	300–400	>70	[85]
33	(5 wt.%)CuO*_x_*/La_2_Ce_2_O_7_	Impregnation, calcination, 600 °C, air, 1 h	0.05 vol.% NH_3_, 5 vol.% O_2_, N_2_ balance, GHSV 20,000 h^−1^	275–425	>80	[67]
34	(10 wt.%)Ce/(2 wt.%)V/TiO_2_	Impregnation, calcination, 400 °C, air, 4 h; pre-treatment conditions: 8 vol.% O_2_/N_2_, 400 °C, 0.5 h	0.02 vol.% NH_3_, 8 vol.% O_2_, 6 vol.% H_2_O, N_2_ balance, GHSV 120,000 h^−1^	300–350	>90	[95]
35	Ce_0.4_Zr_0.6_O_2_	Surfactant-templated method, calcination, 550 °C, air, 3 h	0.1 vol.% NH_3_, 10 vol.% O_2_, He balance, GHSV 40,000 h^−1^	360–380	>90	[93]
36	(6 wt.%)Cu-Ce-Zr *n*(Si)/*n*(Al) = 4	Sol-gel method, calcination, 450 °C, air, 3 h	0.1 vol.% NH_3_, 10 vol.% O_2_, He balance, GHSV 40,000 h^−1^	230	>90	[97]
37	CuO-Fe_2_O_3_ *n*(Cu)/*n*(Fe) = 1:1	Sol-gel method, calcination, 500 °C, air, 4 h	0.08 vol.% NH_3_, 5 vol.% O_2_, Ar balance, GHSV 60,000 h^−1^	225–300	>80	[87]
38	CuO-Fe_2_O_3_ *n*(Cu)/*n*(Fe) = 5:5	Sol-gel method, calcination, 400 °C, air, 4 h	0.08 vol.% NH_3_, 5 vol.% O_2_, Ar balance, GHSV 60,000 h^−1^	250–300	>80	[86]
39	CuFe_2_O_4_ (8.59 wt.% Cu, 7.45 wt.% Fe)	Hard-template method, 600 °C, air, 6 h	0.1 vol.% NH_3_, 0.2 vol.% O_2_, He balance, GHSV 35,000 h^−1^	350–600	>75	[88]
40	CuO-CeO_2_ *n*(Cu)/*n*(Ce) = 6/4	Coprecipitation, calcination, 500 °C, air, 4 h	0.1 vol.% NH_3_, 4 vol.% O_2_, 12 vol.% H_2_O, He balance, WHSV 92,000 mL h^−1^ g^−1^	400	82	[99]
41	CuO-CeO_2_ (10 wt.% Cu)	Surfactant templated method, 500 °C, air, 3 h	0.1 vol.% NH_3_, 10 vol.% O_2_, He balance, GHSV 40,000 h^−1^	250–300	>90	[100]
42	(1 wt.%)Cu-PILC-Verm (Alumina pillared vermiculites)	Ion-exchange, calcination, 450 °C, air, 3 h	0.5 vol.% NH_3_, 2.5 vol.% O_2_, He balance, WHSV 24,000 mL h^−1^ g^−1^	500–550	>95	[89]
43	(5.7 wt.%)Fe-PILC-Phlog (Alumina pillared phlogopite)			500–550	>70	
44	(0.59 wt.%)Cu-PCH (Porous clay heterostructures)	Ion-exchange, 450 °C, air, 3 h	0.5 vol.% NH_3_, 2.5 vol.% O_2_, He balance, WHSV 24,000 mL h^−1^ g^−1^	500–550	>90	[53]
45	(1.43 wt.%)Cu-PCH (Porous clay heterostructures)			400–550	>90	[90]
46	Cu/attapulgite (5–10 wt.% Cu)	Impregnation, 400 °C, air, 4 h	0.005 vol.% NH_3_, 4 vol.% O_2_, N_2_ balance, GHSV 150,000 h^−1^	450–500	>75	[92]
47	natural manganese ore	Fluidization, 12 h	0.05 vol.% NH_3_, 3 vol.% O_2_, He balance, GHSV 15,000–80,000 h^−1^	240	>70	[103]
48	MnO_2_	Calcination, 400 °C, air, 2 h		210	>60	
49	Cu-Mn/TiO_2_ *n*(Cu)/*n*(Mn) = 20/80	Impregnation, rotary evaporator, calcination, 550 °C, air, 2 h	0.06 vol.% NH_3_, 6 vol.% O_2_, N_2_ balance, WHSV 200,000 mL h^−1^ g^−1^	307	not shown	[104]
50	MnO*_x_*-TiO_2_ (27.8 wt.% Mn)	Sol-gel method, calcination, 500 °C, air, 4 h	0.05 vol.% NH_3_, 5 vol.% O_2_, He balance, WHSV 240,000 mL h^−1^ g^−1^	200–350	>60	[106]
51	SmMn_2_O_5_	Organic solution combustion methods, 700 °C, air, 8 h	0.05 vol.% NH_3_, 10 vol.% O_2_, N_2_ balance, WHSV 120,000 mL h^−1^ g^−1^	175–250	>45	[108]
52	(5.0 wt.%)Nb_2_O_5_/SmMn_2_O_5_	Impregnation, 450 °C, air, 2 h		200–250	>60	
53	(30 wt.%)SmMn_2_O_5_/Cu-SAPO	Grinding the mixture; * after hydrothermal aging treatment conditions: 21 vol.% O_2_, 10 vol.% H_2_O, N_2_ balance, 800 °C, 5 h	0.05 vol.% NH_3_, 21 vol.% O_2_, N_2_ balance, GHSV 100,000 h^−1^	225–400 * 300–400	>20 * not shown	[109]
54	La*_x_*Sr_1−*x*_MnO_3_	Hydrothermal method, 400 °C, air, 2 h, post-treatment in 3 M HNO_3_	0.05 vol.% NH_3_, 3 vol.% O_2_, N_2_ balance, WHSV 120,000 mL h^−1^ g^−1^	300–450	not shown	[110]

## 4. Conclusions and Future Perspectives

In this short mini-review, we have discussed recent trends, limits and opportunities offered by hydrotalcite-derived mixed metal oxides as opposed to the other transition metal-based catalysts applied in NH_3_-SCO. Although there are relatively several catalytic systems proposed in the literature, at the same time, their systematic investigations and further improvement are scarce. Furthermore, there is a lack of systematic investigations of the reaction mechanisms. The mechanisms of NH_3_-SCO have been explored mainly by the application of in situ DRIFTS and the indication of the characteristic intermediates of the imide, hydrazine or i-SCR mechanism.

Overall, based on our comparison, two transition metal-based catalytic systems can be selected for the preparation of the next-generation catalysts, i.e., the CuMgAl hydrotalcite-derived mixed metal oxides and Cu/Al_2_O_3_. The complete NH_3_ conversion activity between 375–650 °C and N_2_ selectivity above 70% were reached over hydrotalcite-derived mixed metal oxides. Similarly, Cu/Al_2_O_3_ being the most frequently studied catalyst reached full NH_3_ conversion at 350–500 °C with N_2_ selectivity above 75%. Our revision is further supported by our previous study [111], where the activity and N_2_ selectivity in NH_3_-SCO over hydrotalcite-derived CuMgAl (*n*(Cu)/*n*(Mg)/*n*(Al) = 8/63/29, mol.%) mixed metal oxides and (10 wt.%)Cu/Al_2_O_3_ were tested under NH_3_/O_2_/CO_2_/H_2_O/N_2_ conditions by applying ca. 6.5–6.7 g of catalysts. The mixture of the highly dispersed easily reducible copper oxide and bulk copper oxide species allowed for enhanced activity, N_2_ selectivity and stability. Still, further work is needed on the systematic catalysts’ chemical and phase optimization and catalyst tests, including investigations under more applied reaction conditions concerning either reaction mixture composition (NH_3_ concentration of about 100 ppm with O_2_ concentrations of about 10 vol.%; in the presence of CO*_x_*, SO*_x_* and H_2_O) or temperature (up to 600–700 °C), should follow. Furthermore, thermal stability should be tested, as well as catalyst poisoning via the typical components of the lubricating oil for diesel engines (e.g., Ca, Zn, P and S species). A comprehensive understanding of the involved active species, e.g., through operando technologies under realistic working conditions, could facilitate a knowledge-based catalyst optimization to obtain desired NH_3_ slip catalysts.

## Figures and Tables

**Table 1:** Products obtained from the preparation of the CuM(II)M(III)CO_3_ hydrotalcite-like compounds. Reprinted from [25] with permission from Elsevier.

Cations	Cations’ Ratio	Compounds Identified
CuAl	1.0/1.0	Amorphous species
CuZnAl	2.0/1.0/1.0	HT + R
CuZnAl	3.3/1.6/1.0	HT + R
CuZnAl	1.6/0.8/1.0	HT + R
CuZnAl	1.5/1.5/1.0	HT (HT + R)
CuZnAl	1.2/1.2/1.0	HT
CuZnAl	0.8/0.8/1.0	HT
CuCr	1.0/1.0	Amorphous species
CuZnCr	1.5/1.5/1.0	HT
CuCoCr	2.0/2.0/1.0	HT + M
CuCoCr	1.5/1.5/1.0	HT
CuZnCr	1.5/1.5/1.0	HT
CuMgCr	1.5/1.5/1.0	HT
CuMnCr	1.5/1.5/1.0	MnCO_3_ + HT
CuCoZnCr	1.4/0.1/1.5/1.0	HT
CuZnAlCr	3.0/3.0/1.0/1.0	HT
CuZnFe	1.5/1.5/1.0	Au

HT—hydrotalcite-like compounds; M—Cu_2_CO_3_(OH)_2_ (malachite); R—(Cu,Zn)_2_CO_3_(OH)_2_ (Rosasite); Au—aurichalcite.

## Data Availability

Not applicable.
